# Supplementary material for: Budding yeast complete DNA synthesis after chromosome segregation begins
Source: Nat Commun. 2020 May 8;11:2267. doi: 10.1038/s41467-020-16100-3 (PMC7210879; doi:10.1038/s41467-020-16100-3)
Supplement: Supplementary file 1 — Supplementary Information [file 41467_2020_16100_MOESM1_ESM.pdf]

Supplementary Information

Budding yeast complete DNA synthesis after chromosome segregation begins

Ivanova, Maier, Missarova et al.

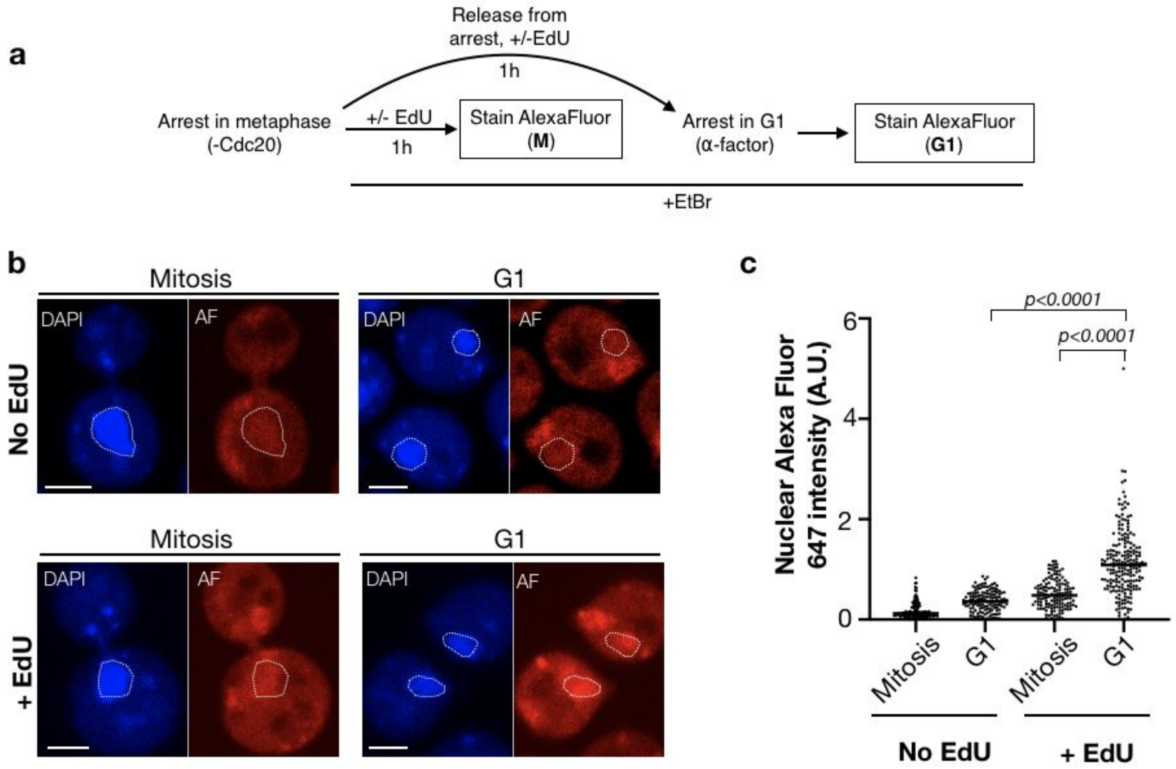

Supplementary Figure 1. **EdU nuclear incorporation in G1 but not M-phase arrested cells. (a)** *MET3pr-CDC20* cells were grown for 3.5 hours in +Met medium to block them in metaphase and treated with EdU as they were either kept in metaphase arrest or released into alpha factor-containing -Met medium for 60 minutes to arrest them in G1. EtBr was added 15 minutes before EdU addition to reduce mitochondrial DNA (see *Methods*). **(b)** Representative cells showing EdU incorporation in G1 but not M phase arrest. **(c)** Cells blocked in G1 showed higher nuclear EdU incorporation than metaphase cells. Number of cells: 118 (No EdU, M); 142 (No EdU, G1); 140 (+EdU, M); 206 (+EdU, G1). *p* values shown correspond to two-sided unpaired t-test without multiple testing correction. This experiment was repeated two times independently with similar results. Scale bars: 2 μm.

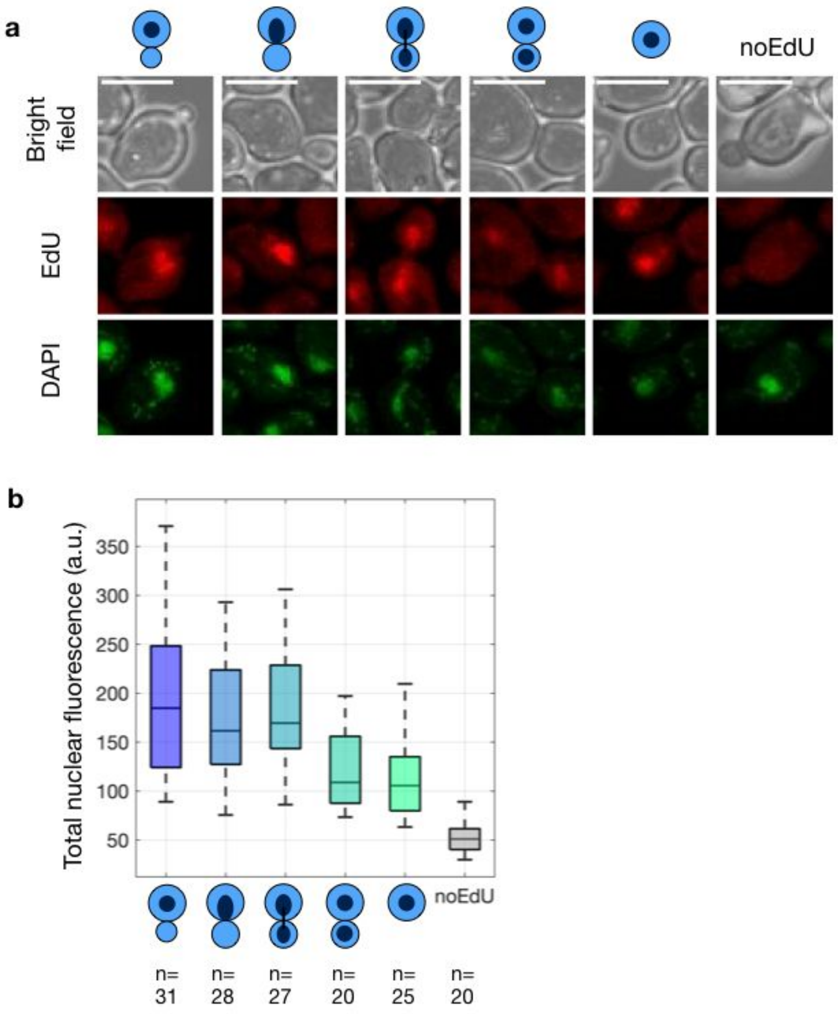

Supplementary Figure 2. **Detection of EdU is not affected by cell cycle stage.** Cells were released from an α-factor block and incubated in EdU for 10 minutes as they entered S phase. After EdU washout, cells were fixed every 15 minutes for the next hour and stained with DAPI in order to define morphological state and measure nuclear EdU incorporation. (a) Cells were split into categories based on their morphological state. (b) Nuclear EdU signal (mean EdU signal multiplied by nuclear area) across cells is equivalent in all cell cycle stages. Note that the decreased signal in cells with divided nuclei as a result of nuclear division. This experiment was repeated two times independently with similar results. Scale bars, 4 μm.

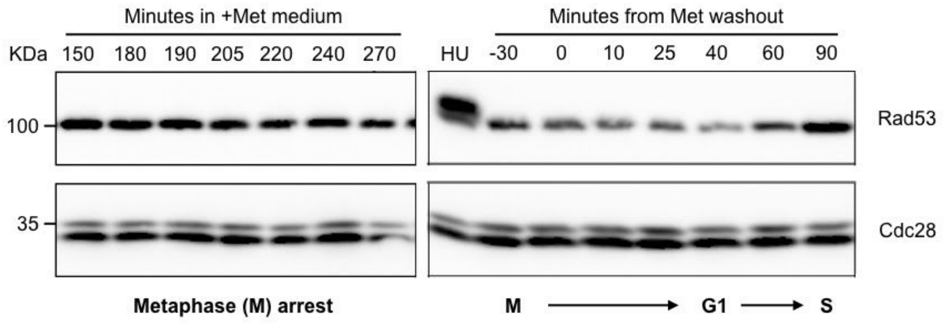

Supplementary Figure 3. No DNA damage during or after release from M phase arrest. Protein extracts were prepared from *MET3pr-CDC20* cells collected at the indicated times relative to addition (left) or washout (right) of 3 mM methionine to block in, and release from, metaphase. Rad53 and Cdc28 (loading control) were detected with specific antibodies (see *Methods*). *HU*, asynchronous culture treated with 0.1 M of HU for 120 minutes, showing Rad53 phosphorylation indicative of DNA damage. Experiments were repeated at least twice with similar results.

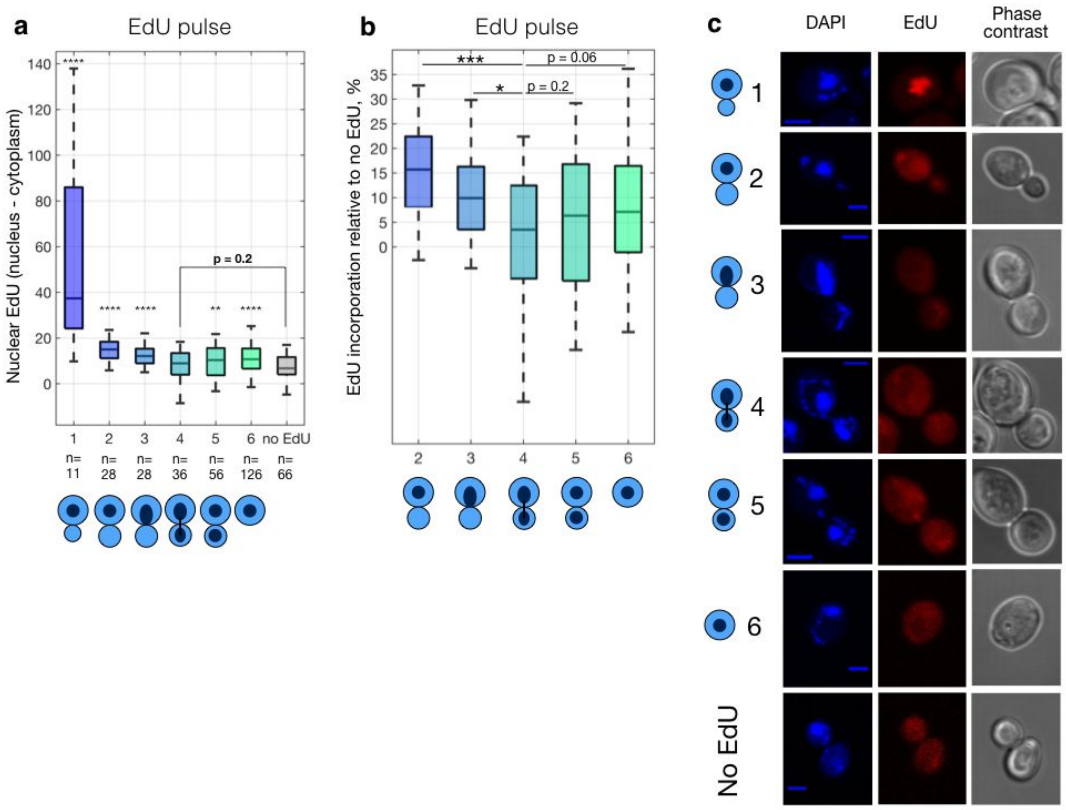

Supplementary Figure 4. Freely cycling cells show nuclear EdU incorporation above background levels in all phases except in early mitosis. Cells growing in mid-log phase were treated with α-factor for 30 minutes to prevent G1 cells from entering S phase, and were incubated in EdU + α-factor for an additional 10 minutes. Additionally, cells were stained with DAPI in order to define morphological state and measure nuclear EdU incorporation. Cells were split into 6 categories based on their morphological state. (**a**) Absolute EdU signal (calculated as average EdU signal in defined by DAPI nuclei area minus average EdU signal in the background (DAPI free)) across cells is statistically higher than in control cells (that were treated with Alexa 647 but not EdU) in all morphological stages except stage 4 (*p < 0.05; *** p < 0.001, two-sided Student T-test). Cells in stage 4 (in which nuclei are segregating between mother and daughter) show no difference in EdU nuclear signal when compared to cells with no EdU. (**b**) Same data as in (a) linearly rescaled so that the mean value for S-phase cells is 100%, and the mean value for no EdU cells is 0%. * < p. 0.05; **p < 0.01; **** p < 0.0001, two-sided Student T-test. (**c**) Examples of DAPI, EdU and bright field images for cells in different morphological states. This experiment was repeated two times independently with similar results. Scale bars, 2 μm. p values: (a) 1 - no Edu: p = 2.8e-14. 2- no EdU: p = 3.5e-09. 3 - no EdU: p = 1.1e-05. 4 - noEdU: 0.207. 5 - noEdU: 0.0134. 6 - noEdU: 2e-05; (b) 2-4: p = 3.5e-04, 3-4: p= 0.0187, 5-4: p = 0.2093, 6-4: p = 0.0576.

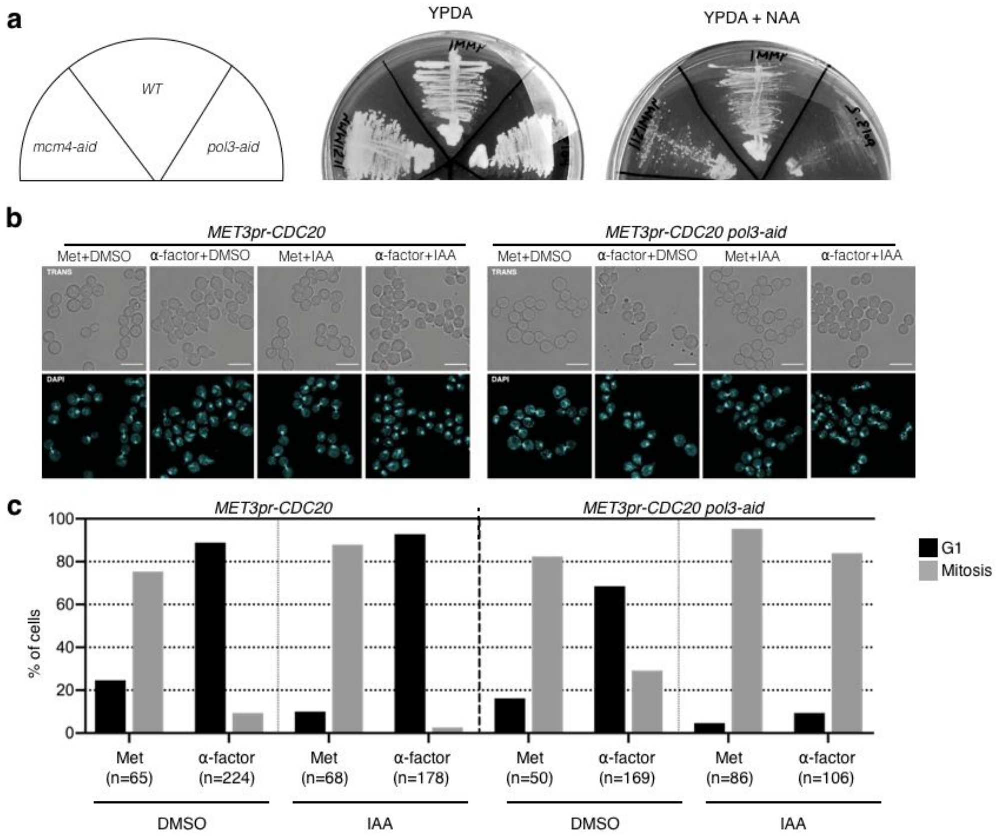

Supplementary Figure 5. Pol3 depletion in M phase inhibits transition into G1. (a) Wild-type and *pol3-aid* cells grown in the absence and presence of NAA (auxin) for 3 days days at 25 ºC to deplete auxin-dependent degron (aid) tagged proteins. *mcm4-aid* serves as positive control. (**b-c**) *MET3pr-CDC20* and *MET3pr-CDC20 pol3-aid* cells were grown for 3 hours in +Met medium to block them in metaphase. Cells were then treated with DMSO or with the auxin IAA (to deplete Pol3). After 60 minutes, cells were either transferred to fresh +Met medium to keep them in metaphase (Met) or released into alpha factor-containing, Met-free medium to arrest them in G1 (α-factor). After 60 minutes, cells were fixed, stained with DAPI, and imaged to determine their cell cycle stage. *MET3pr-CDC20* cells were arrested in metaphase in Met media, and released from metaphase after washout, in either DMSO- or auxin-treated samples. In contrast, *MET3pr-CDC20 pol3-aid* cells were arrested in metaphase in either DMSO or IAA, but failed to release from metaphase specifically when treated with auxin. This experiment was repeated three times independently with similar results. Scale bars, 10 μm.

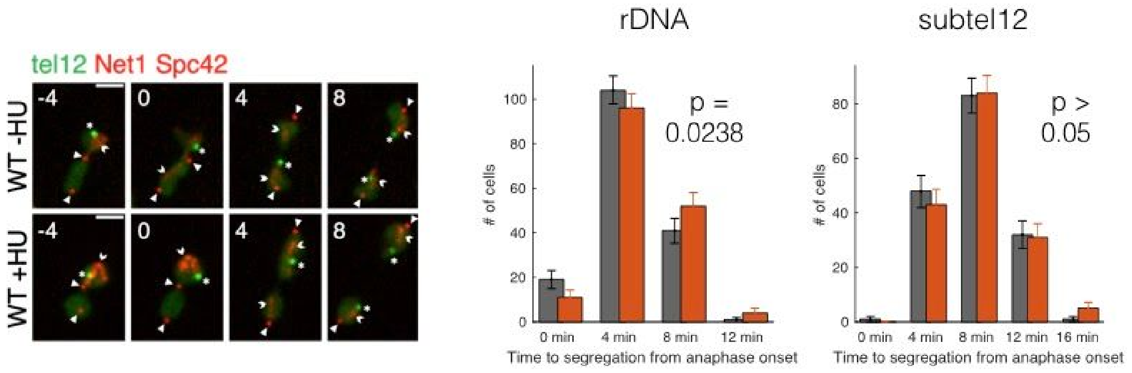

Supplementary Figure 6. Effect of HU treatment in mitosis on segregation of rDNA and tel12R. Cells arrested in metaphase by treatment with nocodazole and released from metaphase in fresh medium (-HU), or treated with 0.1 M HU for 30 minutes and released from the metaphase block in fresh medium containing HU (+HU). A subtelomeric locus (subTel12R) is labeled with TetR-YFP, the rDNA is labeled with Net1-mCherry, and spindle pole bodies are labeled with Spc42-mCherry. Images were acquired every 4 minutes. The time relative to imaging start is indicated in minutes. The time of segregation of the rDNA and subTel12R relative to anaphase onset was determined for n= 163 cells (HU) and n=165 cells (untreated) examined over 2 independent experiments. Error bars are the standard deviation of the mean obtained by bootstrapping, p values are from a two-sided two-sample t-test. Scale bars, 2 μm.

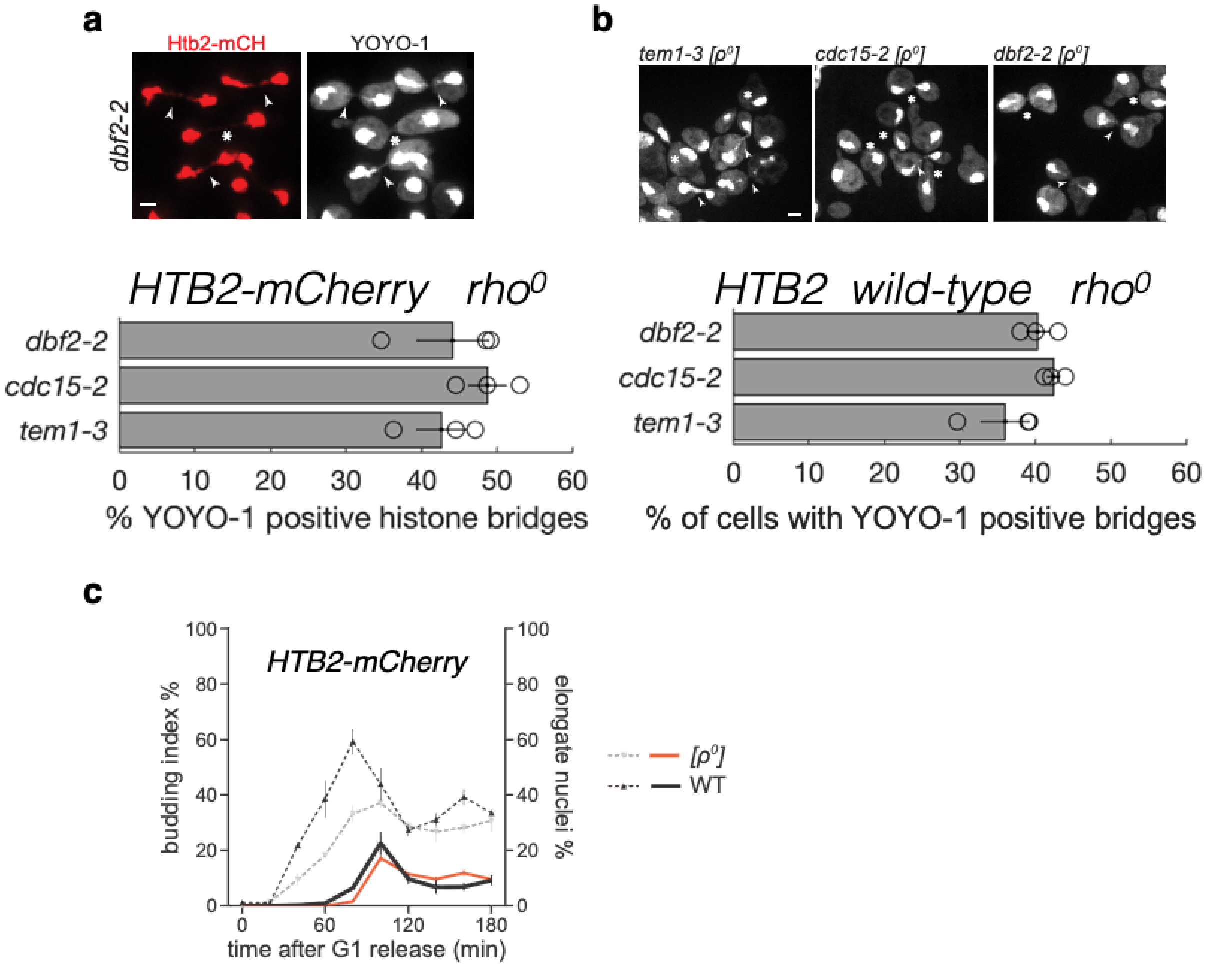

Supplementary Figure 7. Htb2-mCherry bridges have DNA. (a) MEN mutants expressing Htb2-mCherry were arrested and stained with YOYO-1, and cells with Htb2-mCherry bridges scored to see if they contain a YOYO-1 signal that overlaps the chromatin bridge. n= 307 (*tem1*), 314 (*cdc15*), and 318 (*dbf2*) cells examined over 3 independent experiments. **(b)** YOYO-1 staining in *HTB2+* cells. Strains lack mitochondrial DNA [rho-] that would interfere with the YOYO-1 nuclear DNA signal. n= 256 (*tem1*), 316 (*cdc15*), and 308 (*dbf2*) cells examined over 3 independent experiments. **(c)** [rho-] cells do not have anaphase bridges. WT cells with and without mitochondrial DNA released from a G1 block, both strains contain Htb2-mCherry, one is petite, one isn't. Note that the petite strain is the starting strain for the petite MEN mutants. More than 100 cells were counted per time point and replicate. Mean and SEM of three independent experiments are shown. Rho- strains were isolated by picking small colonies from a streak of the parent strain, confirmed by lack of growth on glycerol and by weak or absent mitochondrial DNA staining using Hoechst. Scale bars: 2 μm.

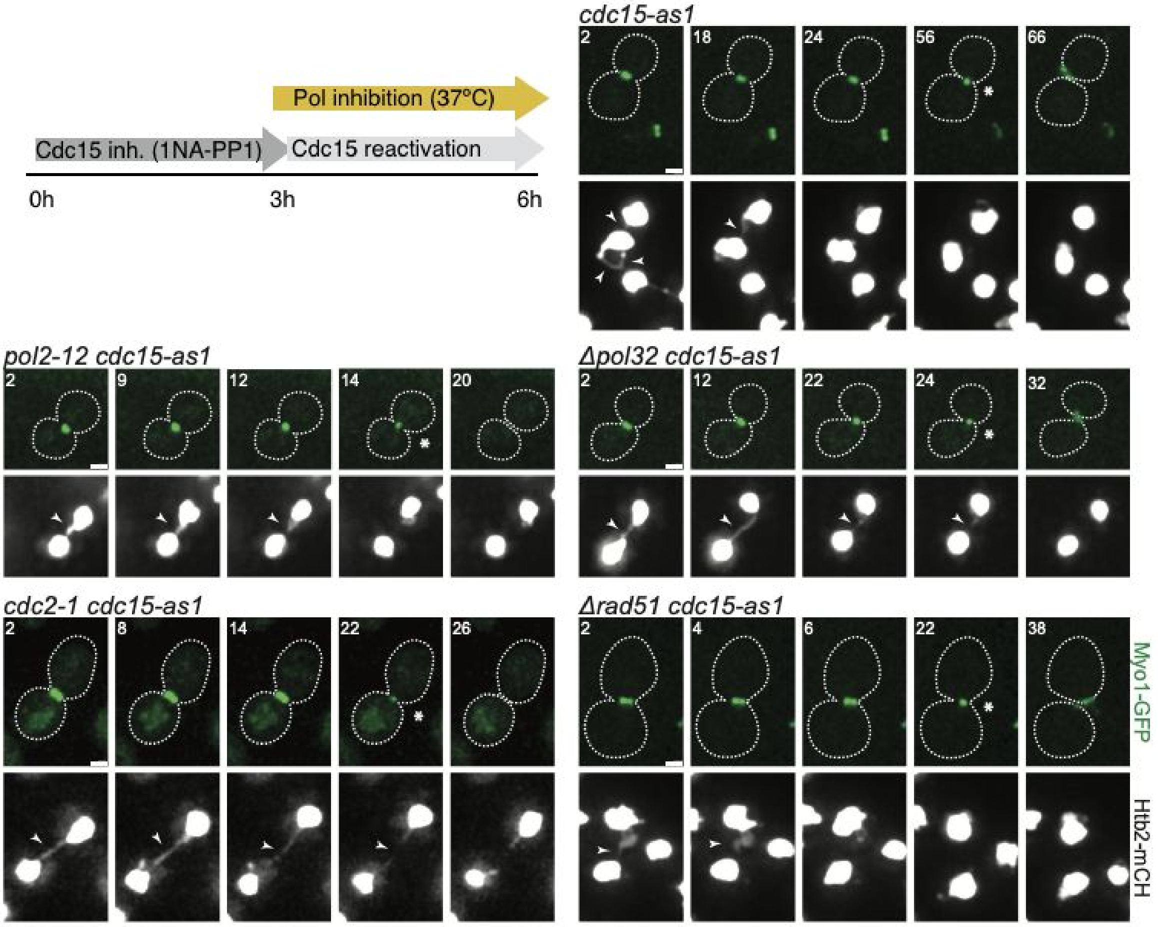

Supplementary Figure 8. DNA polymerases are required for chromatin bridge resolution after MEN reactivation. Examples of cells quantified in figure 5c. 1NA-PP1 was added to mid-log phase cultures at 25 ºC to inactivate Cdc15. After 3 hours, 1NAA-P1 was removed to allow Cdc15 reactivation, cultures were shifted to 37ºC, and cells imaged by time-lapse fluorescence microscopy. t=0 corresponds to the start of imaging (temperature shift). Arrowheads point to chromatin bridges and asterisks mark cytokinesis (actomyosin ring contraction). Whereas *cdc15-as1* and *cdc15-as1 rad51Δ* cells resolve their bridges before cytokinesis, inactivation of Pol2, Pol32 or Pol3 (*cdc2-1*) leads to bridge disappearance during cytokinesis (cut phenotype). These experiments were repeated three times with similar results. Scale bars: 2 µm.

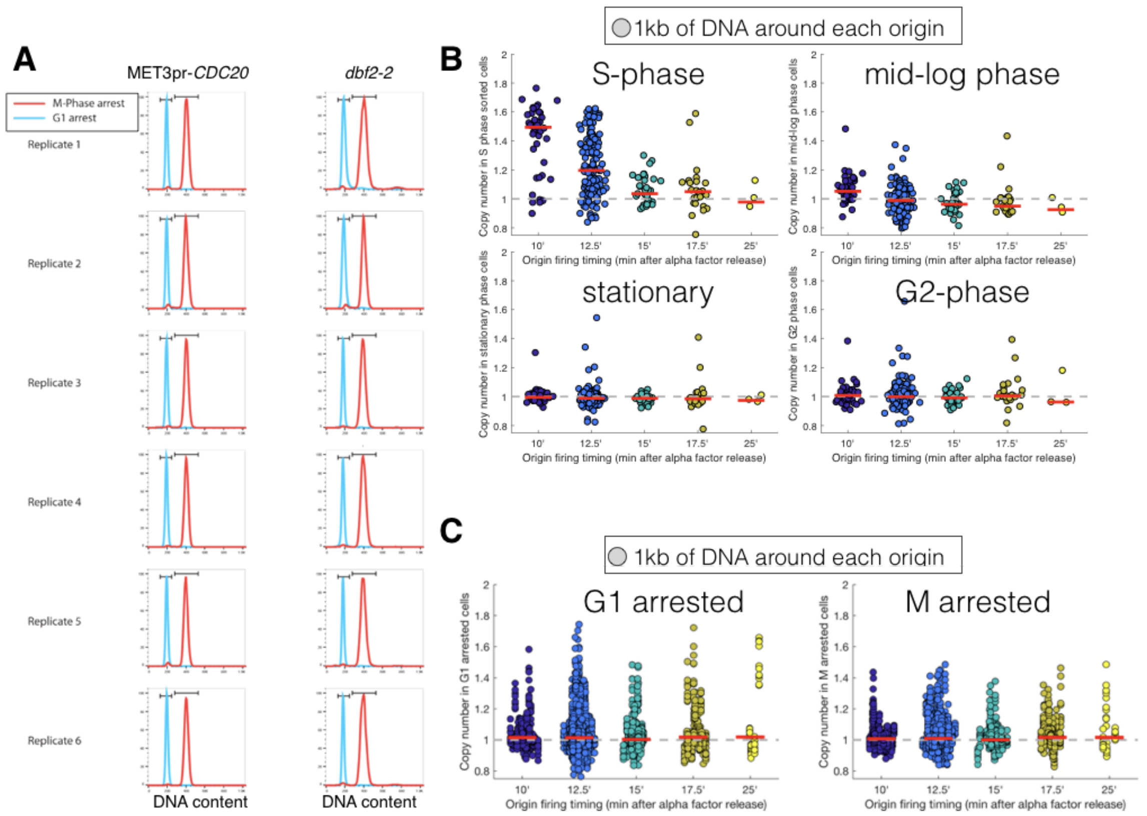

Supplementary Figure 9. Synchrony measurements and tests for contamination from S-phase cells. (A) Flow cytometry profiles from sequenced MET3pr-*CDC20* and *dbf2-2* strains to accompany Fig 3A. Each plot shows the two samples resulting from a split culture. In blue, the G1 arrest at the start of the experiment when the culture was split into two. In red, the metaphase or anaphase arrest 3 hrs after release from G1. Microscopy imaging of DAPI-stained cells was used to independently assess the fraction of cells in G1 (unbudded, mononucleated) and either metaphase or late anaphase (large budded cells with one or two nuclei, respectively) in each sample (>95% for all samples, n>100 cells). **(B)** Each point is the measured copy number from the 1 kb region surrounding a single origin of replication that fires at a given time after an alpha-factor release (data from [1]). Contamination of S-phase cells in the arrested population would lead to a higher copy-number around the early firing origins (20% early S-phase cells would result in a copy-number of 1.2 at all early firing origins). Red lines show the median copy number in each group. **(C)** Control analysis for (B) using data from [2] showing copy-number around early-firing origins for 1N and 2N cells.

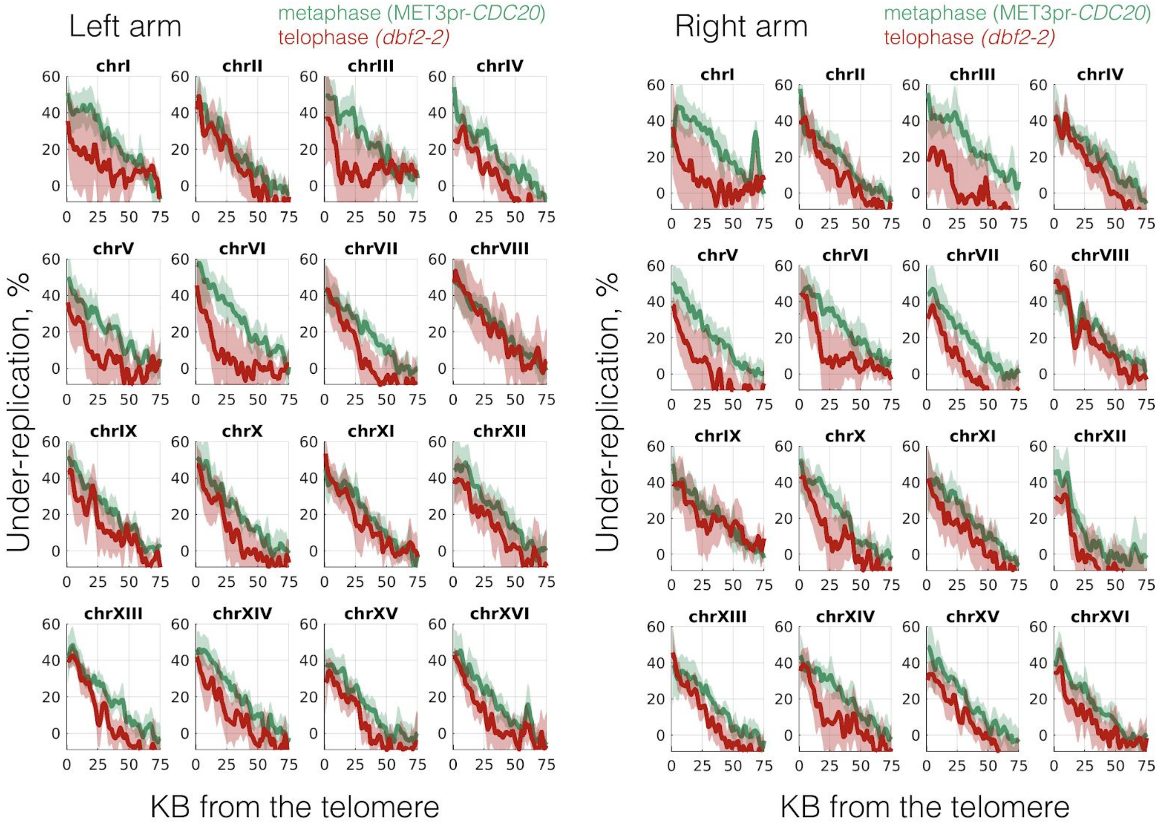

Supplementary Figure 10. Under-replication is different at different chromosome arms, but shows a similar trend across all arms. Under-replication for all subtelomeric regions for metaphase (MET3pr-*CDC20*, in green) and late anaphase (*dbf2-2*, in red) arrests. Shadows correspond to standard deviation across biological replicates (6 for MET3pr-*CDC20*, 5 for *dbf2-2*).

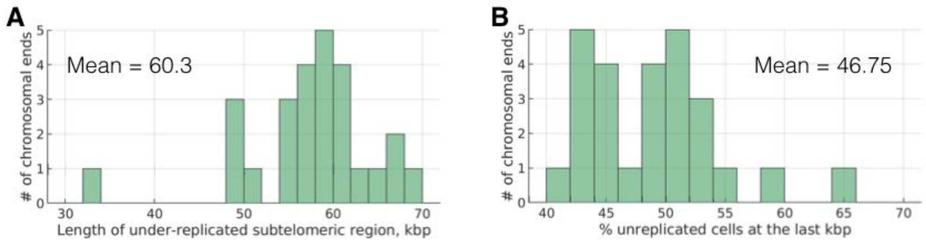

Supplementary Figure 11. The extent of under-replication is different for different chromosomal ends. (A) Distribution of length of significantly under-replicated subtelomeric regions for all 32 chromosomal ends. **(B)** Distribution of percentage of unreplicated cells at the last one kb for all 32 chromosomal ends.

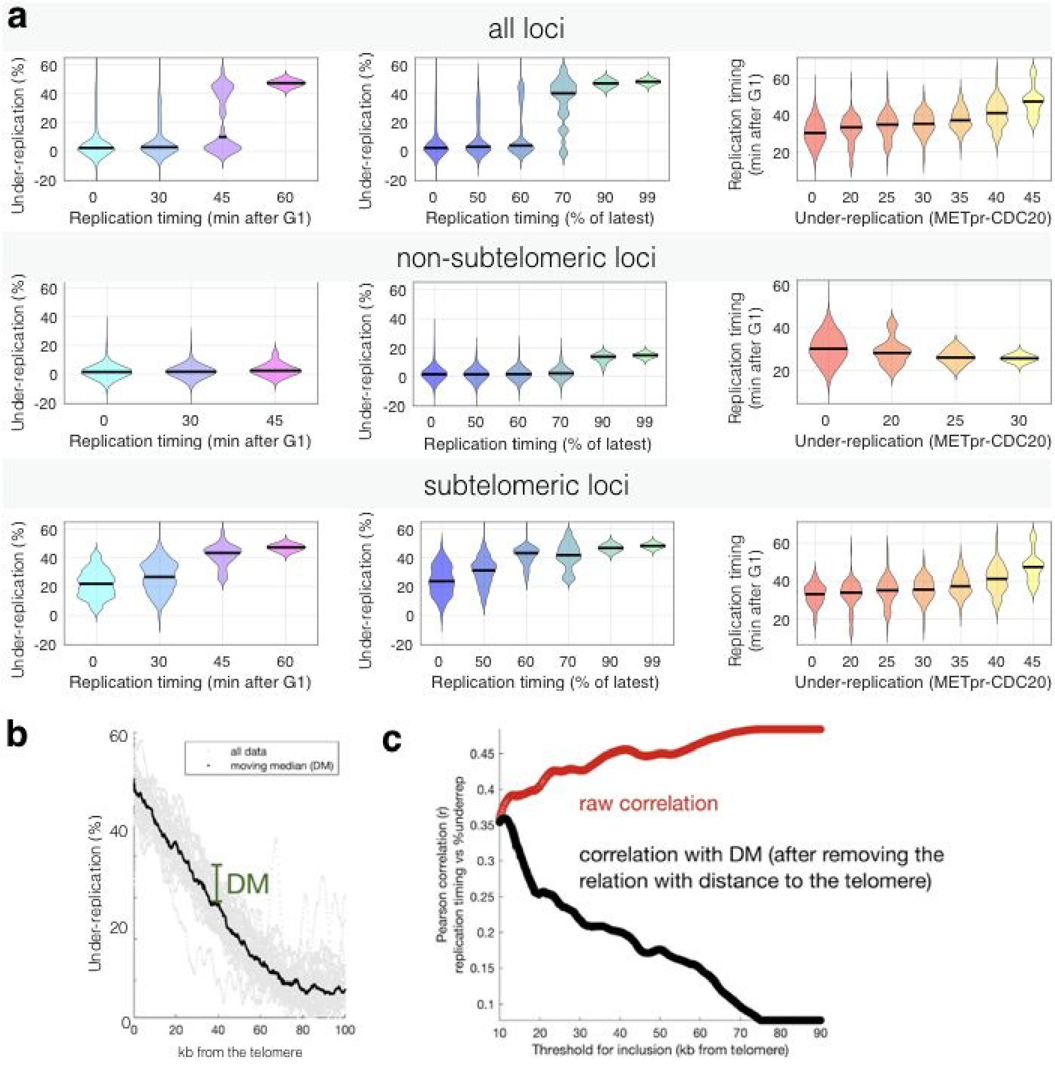

Supplementary Figure 12. Regions that are under-replicated in metaphase are late replicating, but not all late replicating regions are under-replicated. (a) Replication timing for each 200-bp window across all genomic DNA was estimated based on spline interpolation of recorded replication timing (measured in minutes, after release from alpha-factor arrest) from [3]. All genomic loci were split based on their replication timing (blue color-map) or % under-replication (red color-map) bins. **(b)** We used a moving median to determine relation between %underrep and distance to the telomere (black line) and then used correlation with the distance-to-the-median (DM) to ask if %underrep can explain the difference between the measured % under-replication and the moving median value (this method is commonly used for noise in gene expression, eg: Newman et al. 2006, doi:10.1038/nature04785). The resulting 'DM' value (shown in b) is the %underrep after taking into account the distance to the telomere. **(c)** The correlation between %underrep and replication timing for the raw data (red) or for % DM under-replication (black). %underrep-DM is correlated with replication timing in subtelomeric regions.

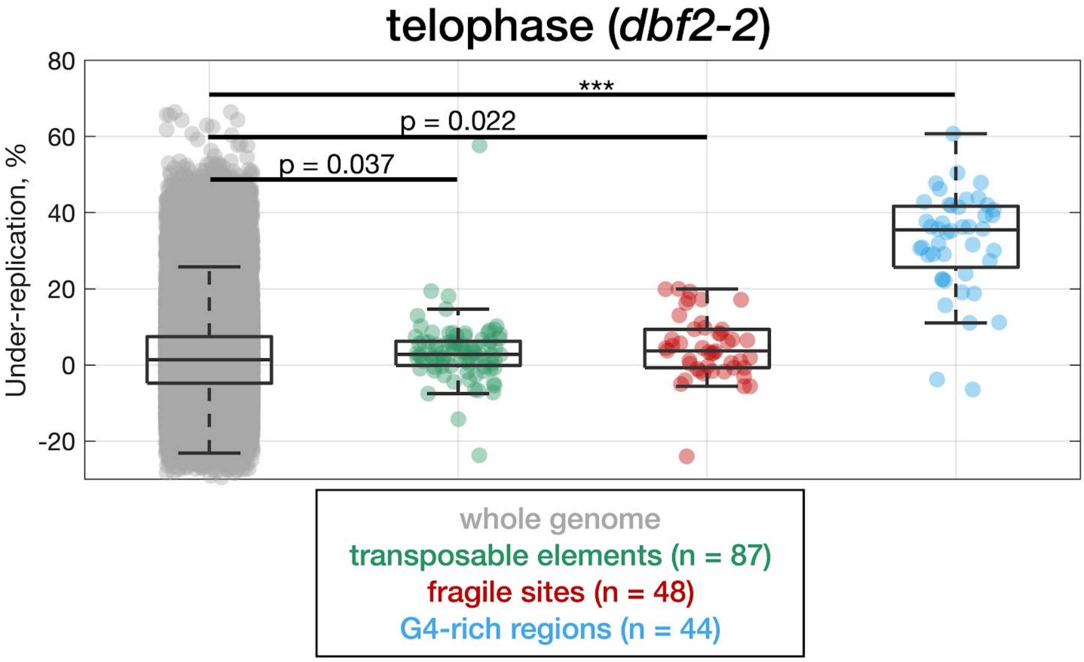

Supplementary Figure 13. Regions with G-quadruplexes are under-replicated in telophase arrested cells. The extent of under-replication for transposable elements (green) and fragile sites (red) is much lower than that of G4-rich regions (and lower than in metaphase-arrested cells, see Figure 6e). Regions with high frequency of G-quadruplexes remain under-replicated (*** $p < 0.0001$, two-sided Wilcoxon rank test).

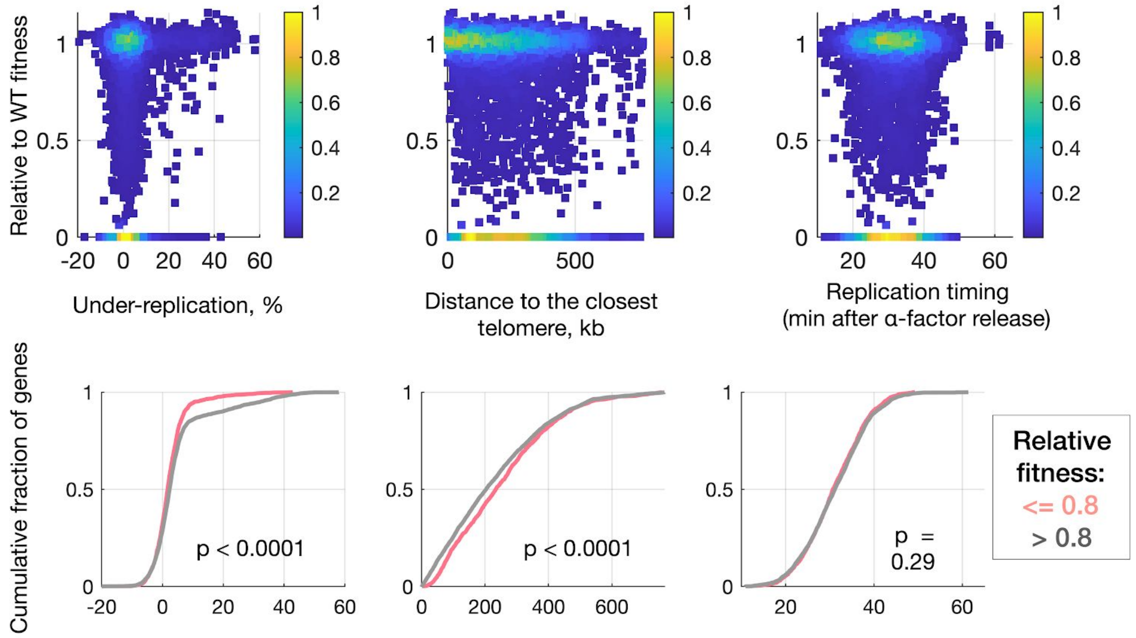

Supplementary Figure 14. Essential genes, and ORFs with relatively high fitness cost (>= 20% once knocked out) have lower under-replication and are further away from telomeres than ORFs with low fitness cost. There is no difference in the distribution of replication timing between these two groups of genes (two-sided Student T-test).

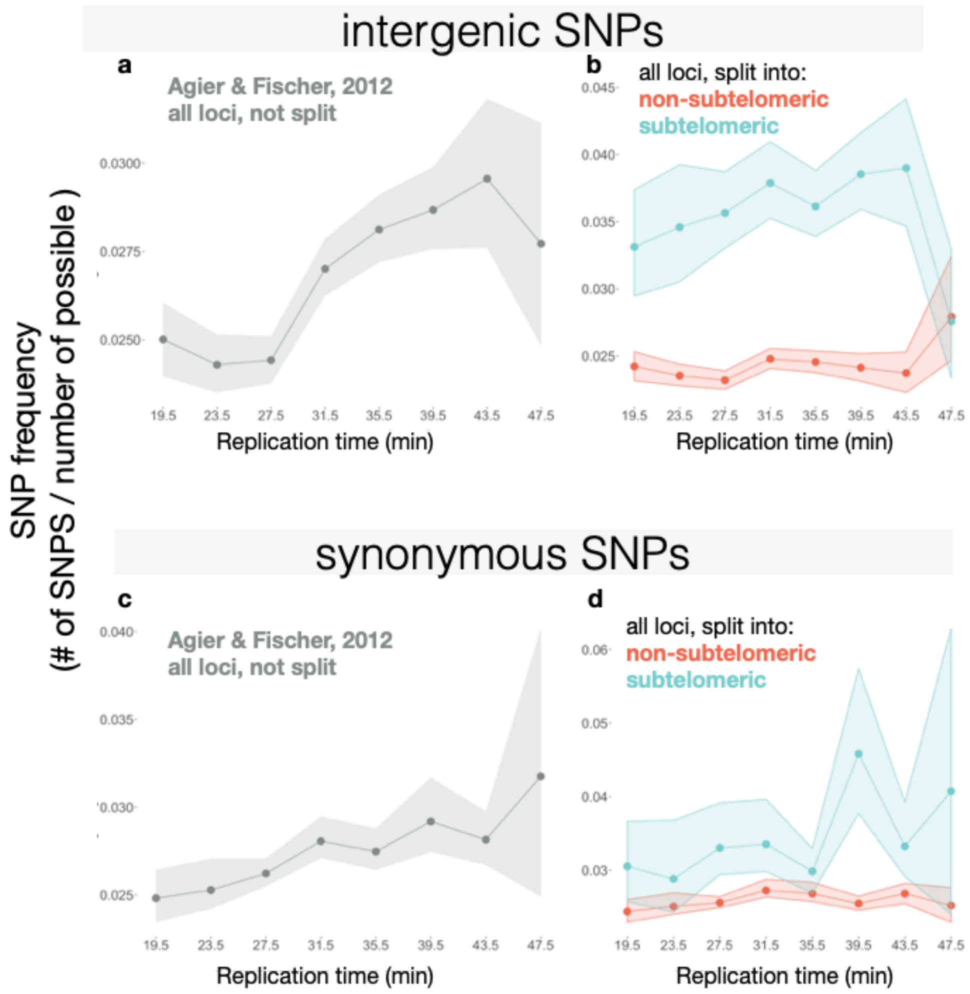

Supplementary Figure 15. Intergenic and synonymous SNP frequencies are higher in subtelomeric regions regardless of their replication timing. Left panels: Intergenic (A) and synonymous (C) SNP frequencies, extracted from [4] are higher for later replicating regions (data from [3]). Rates are calculated per replication time bin (4 minutes). The intergenic frequency is calculated by dividing the total number of intergenic SNPs by the total number of the intergenic nucleotides. The synonymous frequency is calculated by dividing the total number of synonymous SNPs by the total number of synonymous positions. Centers of error bars represent the total number of SNPs calculated across all regions with the corresponding replication timing. Shaded error bars represent the confidence interval at 95% (data is bootstrapped 10000 for each replication time bin). Right panels: The corresponding data are split into subtelomeric (blue) and not-subtelomeric (red): B for intergenic and D for synonymous. Shaded error bars represent the confidence interval at 95% (data is bootstrapped 10000 for each replication time bin).

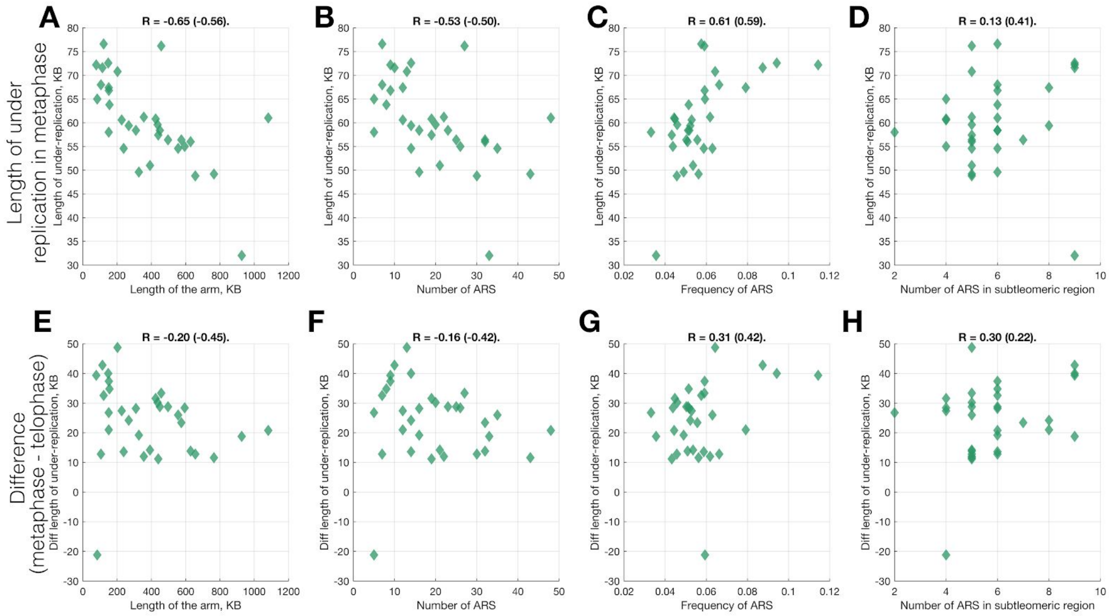

Supplementary Figure 16. Chromosome arms with longer under-replicated regions tend to be shorter and have higher ARS densities. (A-D) For each chromosome arm the length of statistically significant under-replication in metaphase arrested cells (y-axis) is shown plotted against chromosome arm length, the number of ARS elements, the density of ARS elements (per KB), or the number of ARS elements in the subtelomeric 75kb. For panel (A), the correlation of all arms is -0.6471, for the subset of arms with lengths >=100kb, the correlation is -0.6244, and for the subset of arms >= 100kb and < 800kb, and with length of under replication < 65kb, the correlation is -0.5636 (p<-0.01 for all subsets). **(E-H)** For each chromosome arm the difference in the length of statistically significant under-replication (metaphase arrested cells - telophase arrested cells) (y-axis) is shown plotted against the same features as in A-D. For each panel, the Pearson correlation for all 32 arms is shown, and in parenthesis, the correlation excluding the outlier.

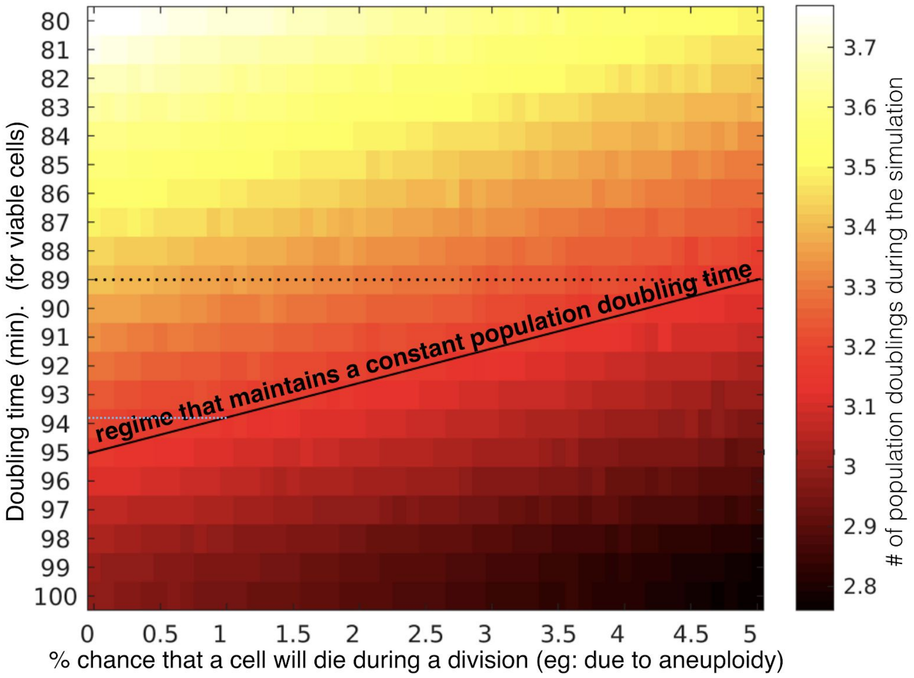

Supplementary Figure 17. A stochastic simulation of the ability of increased population growth to offset an increase in death rate within a clonal population. A population of cells grows and divides with some doubling time (y-axis) and during each division, each cell has a probability of dying (x-axis). Each cell in the matrix is an independent simulation; all simulations were run for the same amount of simulated time, and the increase in population size was calculated as the two parameters (growth rate and death rate) were varied. The dashed blue line shows that if the per-cell doubling time decreases by 90 seconds, the population-level growth rate will be higher, even if the increase in growth rate comes at a cost of 1% of cells dying.

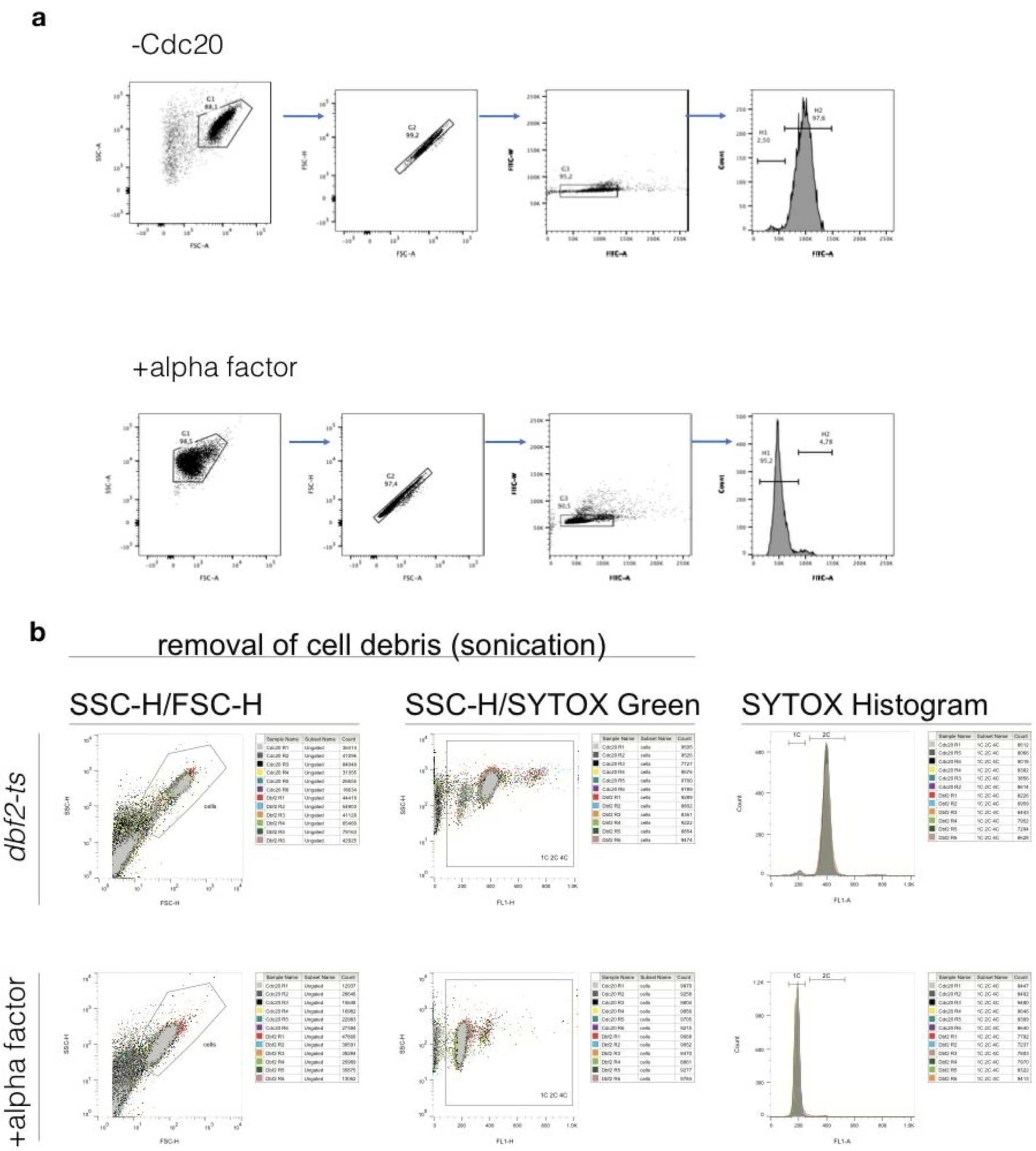

Supplementary Figure 18: FACS gating strategies. Show are the gating strategies for the cells shown in Figure 1b (a) and Supplementary Figure 9a (b). Note the relatively high amount of debris gated out in (b) due to sonication, since MEN mutants tend to clump due to cytokinesis defects.

Supplementary References

1. Alvino, G. M. *et al.* Replication in hydroxyurea: it's a matter of time. *Mol. Cell. Biol.* **27**, 6396–6406 (2007).
2. Müller, C. A. *et al.* The dynamics of genome replication using deep sequencing. *Nucleic Acids Res.* **42**, e3 (2014).
3. Raghuraman, M. K. *et al.* Replication dynamics of the yeast genome. *Science* **294**, 115–121 (2001).
4. Agier, N. & Fischer, G. The mutational profile of the yeast genome is shaped by replication. *Mol. Biol. Evol.* **29**, 905–913 (2012).
