# Supplementary material for: Budding yeast complete DNA synthesis after chromosome segregation begins
Source: Nat Commun. 2020 May 8;11:2267. doi: 10.1038/s41467-020-16100-3 (PMC7210879; doi:10.1038/s41467-020-16100-3)
Supplement: Supplementary file 3 — Description of Additional Supplementary Files [file 41467_2020_16100_MOESM3_ESM.pdf]

Description of Additional Supplementary Files

File Name: Supplementary Data 1
Description: cell cycle arrest of strain used for DNA copy number analysis by genome sequencing

File Name: Supplementary Data 2
Description: Length and extent of subtelomeric under-replication in metaphase and telophase

File Name: Supplementary Data 3
Description: Gene ontology of genes under-replicated in mitosis

File Name: Supplementary Data 4
Description: Strains used in this study
