# Supplementary material for: Budding yeast complete DNA synthesis after chromosome segregation begins
Source: Nat Commun. 2020 May 8;11:2267. doi: 10.1038/s41467-020-16100-3 (PMC7210879; doi:10.1038/s41467-020-16100-3)
Supplement: Supplementary file 8 — Reporting Summary [file 41467_2020_16100_MOESM8_ESM.pdf]

nature research

Corresponding author(s): Lucas B. Carey & Manuel Mendoza

Last updated by author(s): 25/3/2020

Reporting Summary

Nature Research wishes to improve the reproducibility of the work that we publish. This form provides structure for consistency and transparency in reporting. For further information on Nature Research policies, see Authors & Referees and the Editorial Policy Checklist.

Statistics

For all statistical analyses, confirm that the following items are present in the figure legend, table legend, main text, or Methods section.

n/a	Confirmed	
☐	☒	The exact sample size (*n*) for each experimental group/condition, given as a discrete number and unit of measurement
☐	☒	A statement on whether measurements were taken from distinct samples or whether the same sample was measured repeatedly
☐	☒	The statistical test(s) used AND whether they are one- or two-sided<br>*Only common tests should be described solely by name; describe more complex techniques in the Methods section.*
☐	☒	A description of all covariates tested
☐	☒	A description of any assumptions or corrections, such as tests of normality and adjustment for multiple comparisons
☐	☒	A full description of the statistical parameters including central tendency (e.g. means) or other basic estimates (e.g. regression coefficient) AND variation (e.g. standard deviation) or associated estimates of uncertainty (e.g. confidence intervals)
☐	☒	For null hypothesis testing, the test statistic (e.g. *F*, *t*, *r*) with confidence intervals, effect sizes, degrees of freedom and *P* value noted<br>*Give P values as exact values whenever suitable.*
☒	☐	For Bayesian analysis, information on the choice of priors and Markov chain Monte Carlo settings
☒	☐	For hierarchical and complex designs, identification of the appropriate level for tests and full reporting of outcomes
☐	☒	Estimates of effect sizes (e.g. Cohen's *d*, Pearson's *r*), indicating how they were calculated

Our web collection on statistics for biologists contains articles on many of the points above.

Software and code

Policy information about availability of computer code

Data collection	All data were collected using Prism v8, Matlab 2018b, and iQ Live Cell Imaging software (Andor Technology).
Data analysis	All data were analyzed using Fiji/ImageJ and Matlab 2018b

For manuscripts utilizing custom algorithms or software that are central to the research but not yet described in published literature, software must be made available to editors/reviewers. We strongly encourage code deposition in a community repository (e.g. GitHub). See the Nature Research guidelines for submitting code & software for further information.

Data

Policy information about availability of data

All manuscripts must include a data availability statement. This statement should provide the following information, where applicable:

- Accession codes, unique identifiers, or web links for publicly available datasets
- A list of figures that have associated raw data
- A description of any restrictions on data availability

All sequencing data are at NCBI GEO GSE117268, and all other data and code to perform the analyses and generate the figures are at https://github.com/amissarova/Mendoza__ReplicationEvolution

Field-specific reporting

Please select the one below that is the best fit for your research. If you are not sure, read the appropriate sections before making your selection.

☒ Life sciences ☐ Behavioural & social sciences ☐ Ecological, evolutionary & environmental sciences

Life sciences study design

All studies must disclose on these points even when the disclosure is negative.

Sample size	Microscopy: For most experiments, at least 100 cells were counted for each replicate. Each experiment was repeated on at least two different days. Given two populations of cells, and two behaviors (0 and 1), a sample size of 100 cells can detect a difference between the two populations with p<0.05 of more than 5 cells (more than 5%) and with p<0.001 of more than 10 cells (more than 10%) (Fisher's exact test). Sequencing: At least six biological replicates were performed for all sequencing experiments. Six biological replicates for sequencing experiments was chosen because this is twice the minimum number of commonly accepted replicates, and past experience and papers published in other groups shows that 1/5-1/10 sequencing experiments yield bad data.
Data exclusions	Of the six biological replicates for sequencing, one outlier was dropped when the data looked nothing like the other five.
Replication	All experiments were repeated at least two times, on two different days. Many of the microscopy experiments were repeated in two different labs, in two different countries, by two different scientists.
Randomization	n/a. there are no patients, or animals, or subjects.
Blinding	Some of the microscopy experiments were quantified blinded, in that they were counted by someone who did not know the treatment of the individual sample. For other experiments, the investigators were not blinded during data collection and analysis. All experiments were performed without any discrimination. There was no group allocation.

Reporting for specific materials, systems and methods

We require information from authors about some types of materials, experimental systems and methods used in many studies. Here, indicate whether each material, system or method listed is relevant to your study. If you are not sure if a list item applies to your research, read the appropriate section before selecting a response.

Materials & experimental systems

n/a	Involved in the study	
☐	☒	Antibodies
☐	☒	Eukaryotic cell lines
☒	☐	Palaeontology
☒	☐	Animals and other organisms
☒	☐	Human research participants
☒	☐	Clinical data

Methods

n/a	Involved in the study	
☒	☐	ChIP-seq
☐	☒	Flow cytometry
☒	☐	MRI-based neuroimaging

Antibodies

Antibodies used	The primary antibodies used were anti-Cdk1 (PSTAIR) (#06-923, Merck Millipore) at a dilution of 1:2000, and anti-Rad53 ab104232 (Abcam) at a dilution of 1:8000. The primary antibodies used were anti-Cdk1 (PSTAIR) (#06-923, Merck Millipore) at a dilution of 1:2000, and anti-Rad53 ab104232 (Abcam) at a dilution of 1:8000. Blots were developed with anti-rabbit IgG Horseradish Peroxidase conjugate (Thermo Fisher Scientific, Cat. No:31460) diluted 1:20000 using the Supersignal West Femto Maximum Sensitivity Substrate (Thermo Scientific).
Validation	Anti-Cdk1/Cdc2 (PSTAIR) Antibody (06-923 Merk Millipore) is a high quality Rabbit Polyclonal Antibody for the detection of Cdk1/Cdc2/Cdc28 (PSTAIR domain) and according to the manufacturer has been validated for ICC, IP and WB.<br>References:<br>• Complementation used to clone a human homologue of the fission yeast cell cycle control gene cdc2. Lee, M G and Nurse, P. Nature, 327:31-5. 1987<br>• Up-regulation of 14-3-3zeta in lung cancer and its implication as prognostic and therapeutic target. Fan, T; Li, R; Todd, NW; Qiu, Q; Fang, HB; Wang, H; Shen, J; Zhao, RY; Caraway, NP; Katz, RL; Stass, SA; Jiang, F. Cancer research 67 7901-6. 2007<br>• An essential role for Cdk1 in S phase control is revealed via chemical genetics in vertebrate cells. Hochegger, H; Dejsuphong, D; Sonoda, E; Saberi, A; Rajendra, E; Kirk, J; Hunt, T; Takeda, S. The Journal of cell biology 178 257-68. 2007<br><br>Anti-Rad53 Antibody (abcam ab104232) is a Rabbit polyclonal Antibody for the detection of S. cerevisie Rad53 (Rad53 aa 800 to the C-terminus) and according to the manufacturer has been validated for IP and WB<br>References:<br>• Can G et al. Helicase Subunit Cdc45 Targets the Checkpoint Kinase Rad53 to Both Replication Initiation and Elongation Complexes after Fork Stalling. Mol Cell 73:562-573.e3 (2019).<br>• Balint A et al. Assembly of Slx4 signaling complexes behind DNA replication forks. EMBO J 34:2182-97 (2015).<br>• Clerici M et al. Mec1/ATR regulates the generation of single-stranded DNA that attenuates Tel1/ATM signaling at DNA ends. EMBO J 33:198-216 (2014).

Eukaryotic cell lines

Policy information about cell lines

Cell line source(s)	yeast cells
Authentication	during genome sequencing, we confirmed that the strains were correct
Mycoplasma contamination	n/a. yeast.
Commonly misidentified lines (See ICLAC register)	no commonly misidentified cell lines were used in the study

Flow Cytometry

Plots

Confirm that:

- ☒ The axis labels state the marker and fluorochrome used (e.g. CD4-FITC).
- ☒ The axis scales are clearly visible. Include numbers along axes only for bottom left plot of group (a 'group' is an analysis of identical markers).
- ☒ All plots are contour plots with outliers or pseudocolor plots.
- ☒ A numerical value for number of cells or percentage (with statistics) is provided.

Methodology

Sample preparation	cells were fixed and DNA stained with Sytox-Green
Instrument	BD Fortessa
Software	Matlab
Cell population abundance	flow-cytometry was used for analysis of DNA content by SytoxGreen, not for sorting
Gating strategy	n/a. cells were not divided into positive or negative groups

☒ Tick this box to confirm that a figure exemplifying the gating strategy is provided in the Supplementary Information.
